# Impact of COVID-19 vaccination: a global perspective

**DOI:** 10.3389/fpubh.2023.1272961

**Published:** 2024-01-11

**Authors:** Priya Singh, Aditya Anand, Shweta Rana, Amit Kumar, Prabudh Goel, Sujeet Kumar, Krushna Chandra Gouda, Harpreet Singh

**Affiliations:** ^1^Division of Biomedical Informatics, Indian Council of Medical Research, New Delhi, India; ^2^Department of Pediatrics Surgery, All India Institute of Medical Sciences (AIIMS), New Delhi, India; ^3^Centre for Proteomics and Drug Discovery, Amity University Maharashtra, Mumbai, India; ^4^Earth and Engineering Sciences Division, CSIR Fourth Paradigm Institute, Bangalore, India

**Keywords:** COVID-19, vaccination, positivity rate, hospitalization, delta variant

## Abstract

**Introduction:**

The COVID-19 pandemic has caused widespread morbidity, mortality, and socio-economic disruptions worldwide. Vaccination has proven to be a crucial strategy in controlling the spread of the virus and mitigating its impact.

**Objective:**

The study focuses on assessing the effectiveness of COVID-19 vaccination in reducing the incidence of positive cases, hospitalizations, and ICU admissions. The presented study is focused on the COVID-19 fully vaccinated population by considering the data from the first positive case reported until 20 September 2021.

**Methods:**

Using data from multiple countries, time series analysis is deployed to investigate the variations in the COVID-19 positivity rates, hospitalization rates, and ICU requirements after successful vaccination campaigns at the country scale.

**Results:**

Analysis of the COVID-19 positivity rates revealed a substantial decline in countries with high pre-vaccination rates. Within 1–3 months of vaccination campaigns, these rates decreased by 20–44%. However, certain countries experienced an increase in positivity rates with the emergence of the new Delta variant, emphasizing the importance of ongoing monitoring and adaptable vaccination strategies. Similarly, the analysis of hospitalization rates demonstrated a steady decline as vaccination drive rates rose in various countries. Within 90 days of vaccination, several countries achieved hospitalization rates below 200 per million. However, a slight increase in hospitalizations was observed in some countries after 180 days of vaccination, underscoring the need for continued vigilance. Furthermore, the ICU patient rates decreased as vaccination rates increased across most countries. Within 120 days, several countries achieved an ICU patient rate of 20 per million, highlighting the effectiveness of vaccination in preventing severe cases requiring intensive care.

**Conclusion:**

COVID-19 vaccination has proven to be very much effective in reducing the incidence of cases, hospitalizations, and ICU admissions. However, ongoing surveillance, variant monitoring, and adaptive vaccination strategies are crucial for maximizing the benefits of vaccination and effectively controlling the spread of the virus.

## Introduction

Spurred by the spread of new variants of Severe Acute Respiratory Syndrome Coronavirus-2 (SARS-CoV-2), the coronavirus infectious disease 2019 (COVID-19) pandemic has evolved along a unique trajectory. From mid-2020 to late 2021, since the emergence of Alpha to Omicron variants, the world has witnessed the appearance of newer variants of SARS-CoV-2 resulting in exponential spread and increased mortality. In July 2020, the first variant named EU2 (mutation S:447 N) was identified in western Europe, which showed an increased capability to infect (1, 2). Subsequently, several variants of concerns (VOC) were identified such as B.1.1.7 (Alpha) in the UK (September 2020) (3), B.1.351 (Beta) in South Africa (December 2020) (4), P.1 (Gamma) in Brazil (January 2021) (5), and the B.1.617 (Delta) variant in India (January 2021) (6). It is notably evident that the mortality rate due to the COVID-19 disease surged in the countries where the newer variants were discovered (7–10). The B.1.1.7 (Alpha) variant has been linked with an increased risk of transmissibility, hospitalization, and death (8, 11–14). The B.1.351 (Beta) variant was estimated to be 50% more transmissible than all other pre-existing variants (15) and showed increased immune escape capabilities (16). The higher incidence of COVID-19 cases in the younger age groups in the Amazonas (Brazil) may be understood in context of the changes in the pathogenicity of the P.1 variant (17). Initial results have also supported significant increase in fatalities among young and middle-aged individuals with the P.1 (Gamma) mutant (18). A significant rise in the daily infection rate was reported in the state of Maharashtra in India congruent with the appearance of the B.1.617 (Delta) variant (19).

The governments of countries worldwide implemented and followed the non-pharmaceutical interventions (NPI) suggested by the World Health Organization (WHO) to contain the spread of the global pandemic. Yet, the second wave in 2020 unleashed a deadly outbreak globally and emphasized the importance of an effective and reliable vaccination regimen to control the ongoing pandemic during that time. By the year 2021, about 18 vaccines were available globally against COVID-19 which were approved by at least one country. Additionally, there were more than 125 vaccine candidates and numerous ongoing vaccine trials (20, 21). The United Kingdom was the first country to initiate the COVID-19 vaccination program following the emergency use authorization of the Pfizer-BioNTech (22). Other nations also rolled out their vaccination programs in quick succession. With the accelerated pace of vaccine development, several vaccines received approval after demonstrating significant efficacy in respective clinical trials (23, 24).

The mRNA-based Pfizer-BioNTech COVID-19 Vaccine (Comirnaty) and mRNA-1273 (Moderna), the adenovirus-based Gam-COVID-Vac (Sputnik V) and Oxford–AstraZeneca COVID-19 vaccine (marketed as Covishield and Vaxzevria), and the inactivated virus-based Covaxin and Sinopharm COVID-19 vaccine (BIBP vaccine) are among the approved vaccines against COVID-19. With the increasing rates of vaccination and increased availability due to additional authorization of newer vaccines, there is a need to understand the potential impact of vaccination on the COVID-19 positivity rates region wise. Several studies have been reported in the past year on the impact of vaccination on the overall attack rates, hospitalizations, and deaths. Vilches et al. reported Pfizer-BioNTech and Moderna vaccines reduce deaths by 31.5 and 31.9%, respectively, compared to no vaccination (25). The effect of mRNA vaccination in long-term care facility (LTCF) resident have observed and it is indicated that 70% vaccinatinated population resulted in reduced mortality and infection rate by about 75%, while the detectable transmission was reduced by 90% (26). It has been also reported that an mRNA COVID-19 vaccine significantly reduces disease progression to requirements of mechanical ventilation or even death (27). Studies have shown that countries with predominately vaccinated older adult population, predominantly among the older adult population, had a major shift in the age distribution of COVID-19 related deaths (28). These studies consistently support a global pattern of risk reduction among the vaccinated population as compared to unvaccinated populations (27).

Further, it is essential to determine the impact of vaccination so that the viral disease spread and transmission risk of SARS-CoV-2 can be monitored (29). Although the available vaccines significantly reduce the rates of hospitalization, they do not provide complete protection against SARS CoV-2 infection, since several cases of infection in vaccinated individuals have been reported and the effect of vaccination in preventing new infections in the general community is under consideration (22, 30).

The primary objective of this study was to examine the effect of COVID-19 vaccination on the positivity rates, rates of hospitalization, and the requirements of ICU care. The study is performed by considering the fully COVID-19 vaccinated population until 20 September 2021 in various countries.

## Data and methods

### Study design

**Figure 1:**
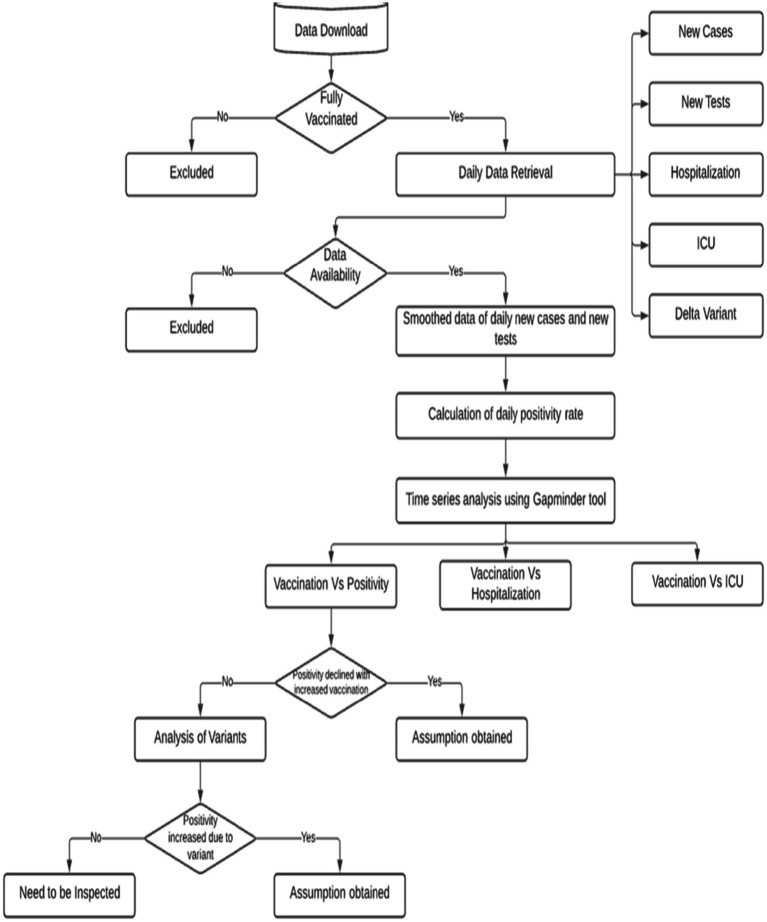
Data analysis workflow.

This is a global retrospective study based on the COVID-19 management data from the first positive case reported until 20 September 2021. The data analysis workflow is shown in Figure 1.

### Data source

The dataset used in the study was obtained from *Our World in Data* (31). Only countries that had completed the second dose of vaccinations were included in the study design. The available data were extracted (on daily basis) for the reported factors, i.e., (i) new COVID-19 cases, (ii) new tests, (iii) new hospitalizations per million, and (iv) new ICU patients per million. The daily data of the Delta variant were collected from the Global initiative on sharing all influenza data (GISAID) database (32) that provides rapid and open access to epidemic and pandemic virus data including genetic sequences and related clinical and epidemiological data associated with the human and viruses.

### Statistical analysis

The primary focus of the study was to analyze the percentage of the fully vaccinated population per 100 in each country. The daily positivity rate, which served as the primary outcome, was defined as the confirmed daily new COVID-19 cases divided by the total number of tests conducted on a particular day in a given country. Additionally, the incidence of the Delta variant was calculated by dividing the number of samples that tested positive for the Delta variant by the total number of samples tested on that specific day in each country. For analysis purposes, the data for the positivity rates, hospitalized cases per million, and ICU hospitalized cases per million were prepared on a daily basis. Time series analysis of daily positivity rates, hospitalized cases, ICU hospitalized cases with respect to daily vaccinations per 100 was performed by using the Gapminder Offline tool (33). The analysis included data from the day when the second dose of vaccination began, extending until the latest available data for each respective country. We have also looked into the incidences of Delta variants across the globe to check whether there is any correlation to the failure in positivity rate reduction for certain countries despite the increased rate of vaccination. Time series analysis of the daily positivity rate in relation to the daily data on Delta variants was conducted using the Gapminder Offline tool (33). To visualize in the graphical user interface, one needs to choose color and size options according to their preferences.

## Results and discussion

**Figure 2:**
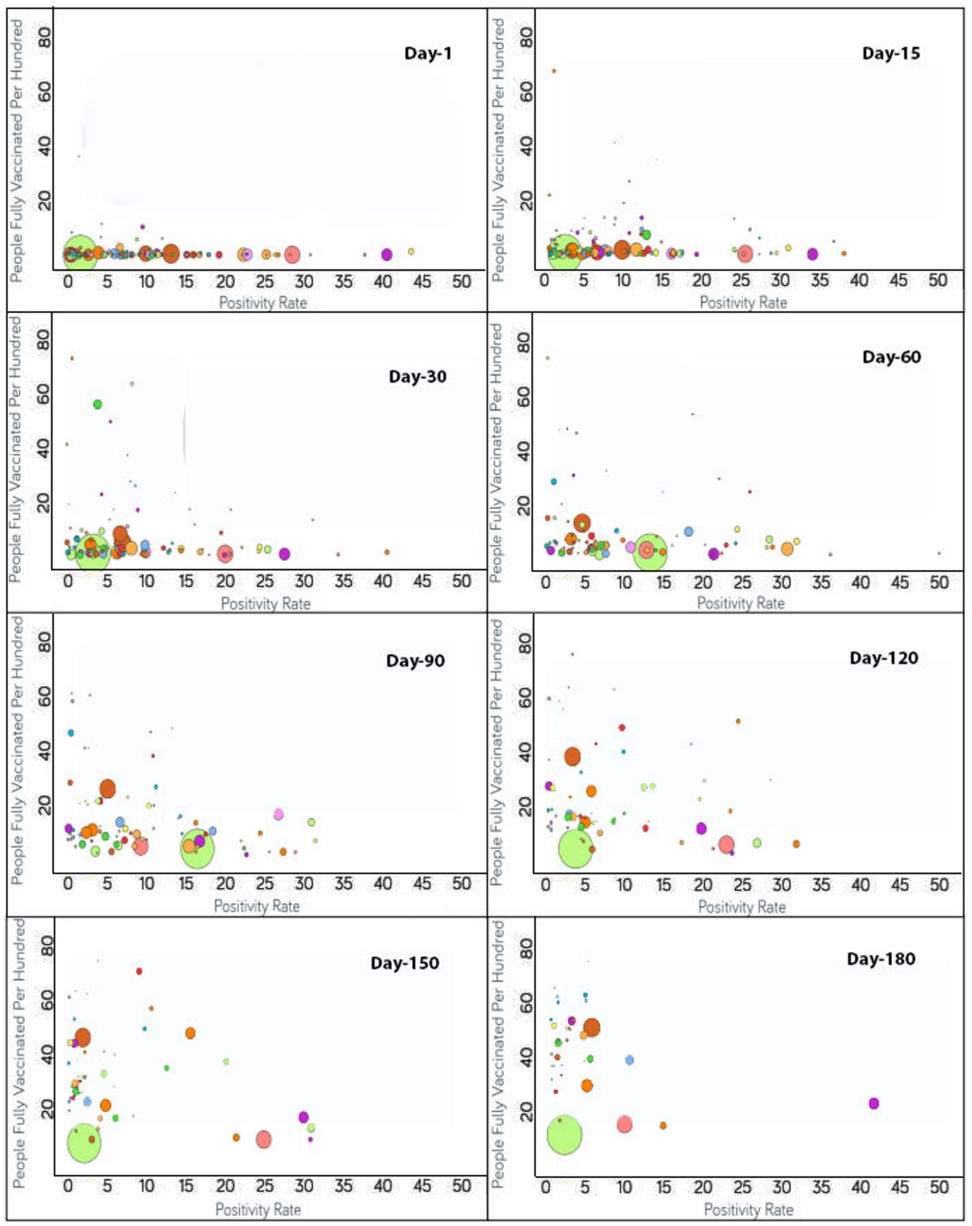
Time series analysis between positivity rates vs. the percentage of the population fully vaccinated per 100 across the world. The X-axis represents the COVID-19 positivity rate with a range of 50 whereas the Y-axis shows the number of fully vaccinated people per 100. Each bubble corresponds to a country with bubble size representing the population. The bubbles are color-coded by country (i.e., fluorescent green: India, rust: USA, purple: United Kingdom, pink: Indonesia, yellow: France, blue: Turkey and so on). The visualization link to generate this figure is https://ccc.icmr.org.in/delta/positivity/.

**Figure 3:**
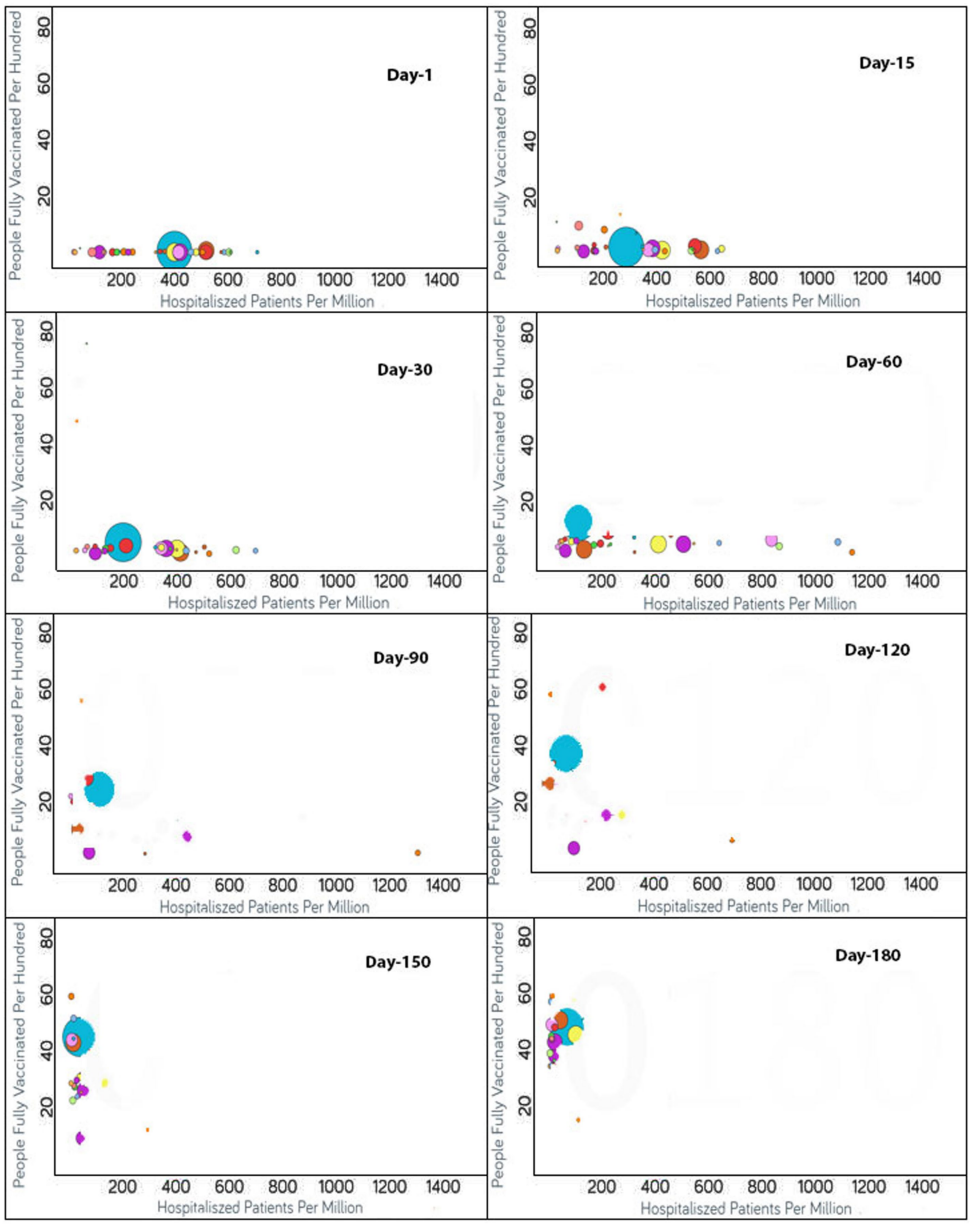
Time series analysis between hospitalized patients per million vs. the percentage of the population fully vaccinated per 100 across the world. The X-axis shows the hospitalization rate per million with a range of 1,400 whereas the Y-axis represents the COVID-19 vaccination rate per 100. Each bubble is a country with the bubble size representing the population. The bubbles are color-coded by country (i.e., blue: USA, purple: Canada, red: Spain, yellow: France, rust: United Kingdom and so on). The visualization link to generate this figure is https://ccc.icmr.org.in/delta/hospitalization/.

**Figure 4:**
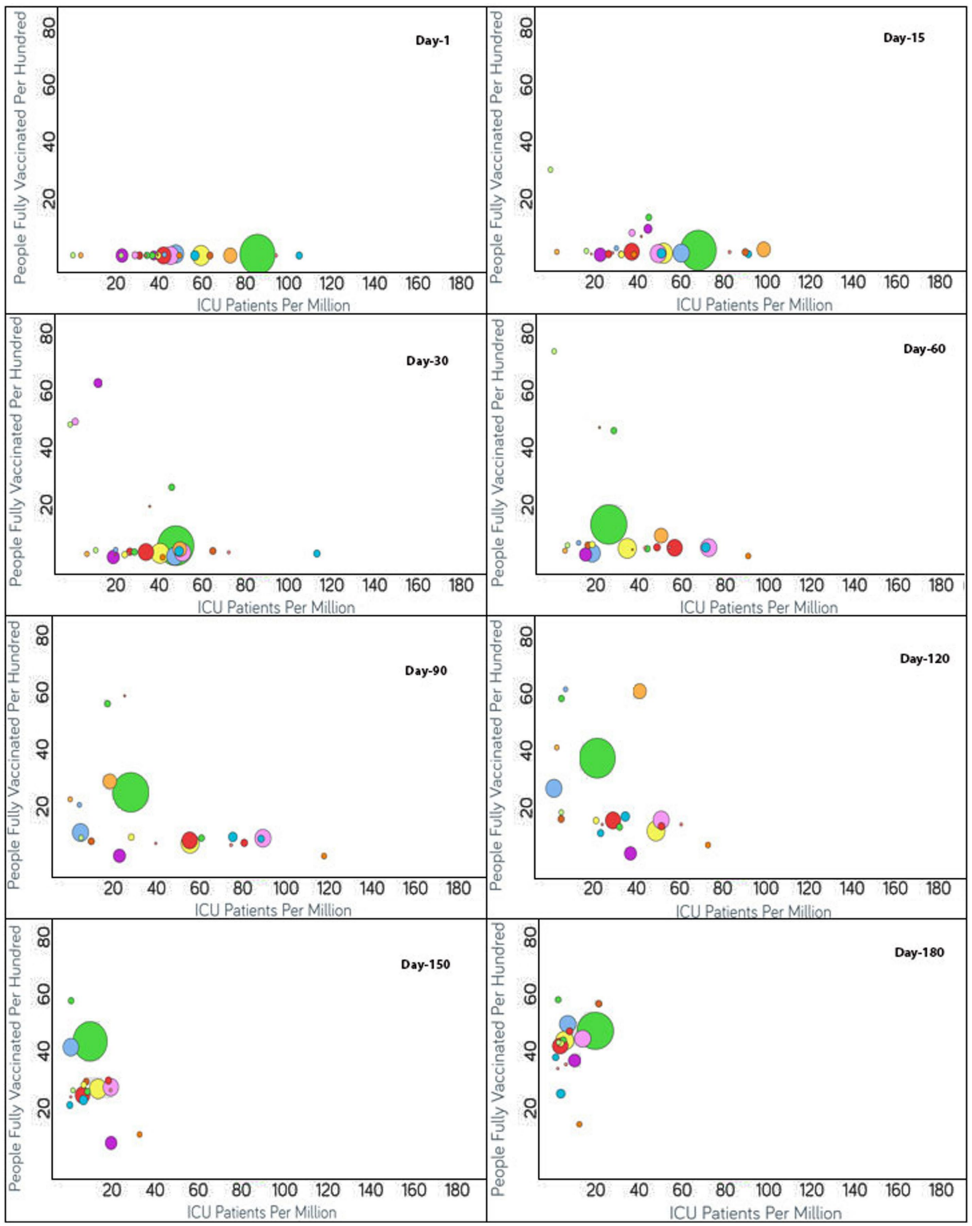
Time series analysis between ICU patients per million vs. the percentage of the population fully vaccinated per 100 across the world. The X-axis represents the ICU patients per million (ranging from 0 to 180) and the Y-axis indicates the persons fully vaccinated per 100 (range 0–80). Each bubble represents a country with its size representing the population and the bubbles are color-coded by country (i.e., green: USA, blue: Czechia, purple: Canada, red: Italy, yellow: Germany and so on). The visualization link to generate this figure is https://ccc.icmr.org.in/delta/icu/.

**Figure 5:**
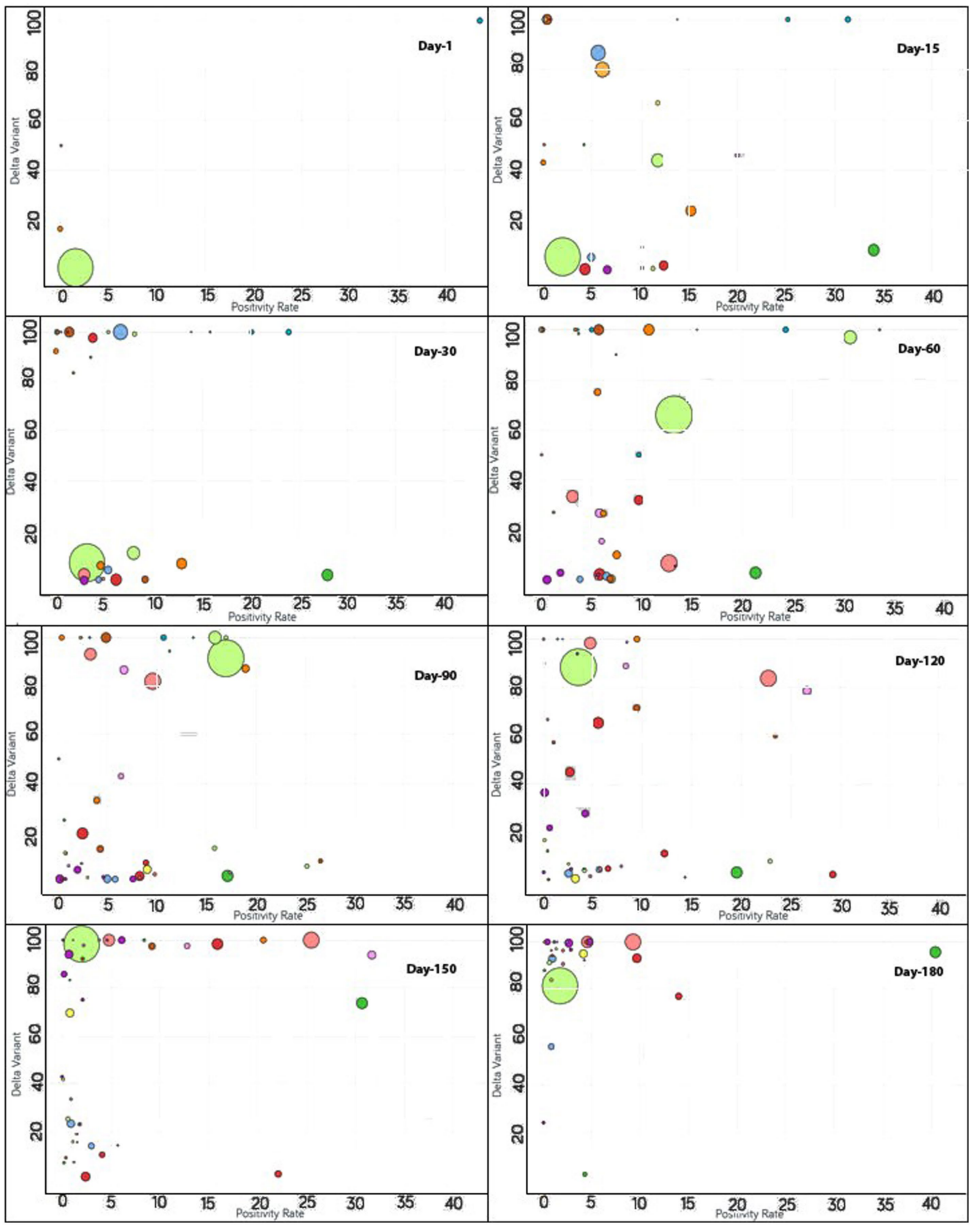
Time series analysis between the Delta variant vs. positivity rates across the world. The X-axis represents the positivity rate and the Y-axis indicates the incidence of the Delta variant. Each bubble represents a country with its size representing the population and the bubbles are color-coded by country (i.e., fluorescent green: India, blue: Pakistan, purple: United Kingdom, red: Japan, yellow: France, pink: Thailand, rust: Vietnam, peach: Indonesia and so on). The visualization link to generate this figure is https://ccc.icmr.org.in/delta/delta/.

To monitor the effectiveness of vaccination, the available data are presented in three parts, with detailed analyses presented in both image and video formats for: (i) fully vaccinated populations vs. Covid-19 positivity rates (Figure 2),^1^ (ii) fully vaccinated populations vs. hospitalizations per million (Figure 3),^2^ and (iii) fully vaccinated populations vs. ICU patients per million (Figure 4).^3^ The findings (as depicted in Figures 2–4) suggest a marginal negative association such that as vaccination rates have increased over time, positivity rates, hospitalizations, and ICU admissions have decreased simultaneously, with a few exceptions noted (Figure 5).^4^ As observed in Figure 5, the positivity rate increased when the Delta variant became a concern in the depicted countries (the United Kingdom, Germany, Italy, Ireland, etc.). However, there may be other reasons for the unexpected rise in the positivity rate (life expectancy, comorbidity like diabetes prevalence, smokers, etc.) that are not accounted for in the present study.

### Vaccination vs. COVID-19 positivity

The analysis of fully vaccinated populations vs. COVID-19 positivity rate is presented in Figure 2, where each bubble represents a country and the bubble size represents the population. The bubbles are color-coded by country. The results obtained indicate that prior to vaccination the positivity rate is fairly high (>30%) for Nepal (44%), Mexico (41%), Paraguay (38%), and Albania (31%). To further analyze the impact of vaccination, countries were divided into three groups based on their positivity rates prior to the vaccination campaigns. The first group comprises countries where the positivity rates were higher than 30% before vaccination [Nepal (44%), Mexico (41%), Paraguay (38%), and Albania (31%)]. Within 1 month of vaccination, the COVID-19 positivity rate was found to be drastically reduced to 0.4% for Albania and in Nepal shrunk by 20% where it reached 24%. For Nepal, the positivity rate further continued to decrease and reached 11% after 90 days of vaccination. Similarly, a lowering of the positivity rate from 41 to 27% was reflected after 30 days of vaccination in Mexico. A further reduction in the positivity rate for Mexico was observed to continue until 110 days of vaccination and hit 16%. However, after 110 days of vaccination, the Delta variant became a concern in Mexico and the positivity rate gradually started increasing. The positivity rate continued to increase until 96 days, and thereafter was observed to be under control since by that time the percentage of the fully vaccinated population reached 27%. For Paraguay, 30 days after vaccination, a slight (4%) decline in the positivity rate was reflected that showed further steady decline reaching down to 2% after 174 days of vaccination.

The second group contains those countries where the positivity rates were between 25 and 30% before vaccination and include Indonesia (29%), Costa Rica (28%), Ecuador (27%), Tunisia (26%), Iran (25%), and Slovenia (25%). For Indonesia, within 90 days of vaccination, the positivity rate declined to 9%. However, after 90 days of vaccination due to the Delta variant effect, Indonesia again witnessed the positivity rate increasing while only 4% population of Indonesia was fully vaccinated at that point. At the 138th day, the positivity rate came under control and started decreasing continuously. For Ecuador, the positivity rate declined consistently from the start of vaccination and reduced to 10% after 150 days of vaccination as the vaccination rate is very high in Ecuador and 54% of the population are fully vaccinated to date. A slight decrease in the positivity rate was observed for Iran (4%) while only 14% of the Iranian population was fully vaccinated. A mild decrease of 14% in the positivity rate was observed in Tunisia after 126 days of vaccination as the vaccination rate was moderate in Tunisia (26%). In Slovenia, the positivity rate declined continuously from the day vaccination started and reduced to less than 2% after 171 days of vaccination since the vaccination rate was high in Slovenia and 46% of the population of Slovenia was fully vaccinated at that time. After 171 days, the positivity rate again showed signs of increment in Slovenia due to the emergence of the Delta variant.

The remaining countries fall under the third group where the positivity rate was below 25% before vaccination. Within the first 15 days, the United Arab Emirates reached close to a 70% vaccination rate. Bangladesh and India had vaccination rates under 3% and the positivity rates showed an increase on days 71 and 81, respectively. From 81 days after vaccination, India started showing a decline in the positivity rate without a noticeable change in the vaccination rate until 112 days. The positivity rate showed an increase for Japan irrespective of the increase in vaccination rate from 111 to 148 days. Thereafter, Japan decreased in positivity rate as the vaccination of the population continued.

### Vaccination vs. hospitalization

The analysis of fully vaccinated populations vs. hospitalized patients per million is presented in Figure 3, where each bubble represents a country and the bubble size represents the population. The bubbles are color-coded by country. In the early stages of vaccination, the hospitalization rates were high, but with the increases in the vaccination rates, the hospitalization rates decreased simultaneously (Figure 3). In the first 15 days after the start of vaccination, the hospitalization rate was above 600 per million for Czechia, Portugal, Slovenia, Spain and Lithuania, where only less than 2% of total population was vaccinated for these countries. Within a span of 30 days, the vaccination rate increased and the hospitalization rate reduced by half for Lithuania, Slovenia, and Spain (Figure 3). The reductions in the rates of hospitalization continued for the next 30 days, but thereafter were interrupted by the emergence of non-parental COVID-19 variants after 60 days of vaccination; thus, a slight rise in hospital rates was observed for Lithuania and Slovenia. But within next few days (i.e., in 20 days), the vaccination rates had increased and the hospitalization rates showed a decline. For Bulgaria, Hungary, and Poland, which were the top three among all countries reported with hospitalization rates above 800, they exhibited a steady surge in hospitalization until 80 days after vaccination. But the rate of hospitalization decreased later when the vaccination rates increased in these countries also. Ninety days after vaccination, almost all the countries reflected a reduction in the hospitalization rates because of the improved vaccinated percentage of the populations. For certain countries that included Croatia, Denmark, Finland, Ireland, Israel, Norway, Portugal, and the UK, the hospitalization rates reduced drastically to below 50 per million. A reduction in hospitalizations to below 200 per million was observed for Austria, Belgium, Canada, Cyprus, Czechia, Estonia, Hungary, Lithuania, Poland, Slovakia, Spain, Switzerland, and the USA. The reduction in hospitalizations continued and it was noticed that after 120 days of vaccination, the hospitalization rates reduced to the point of 200 per million for France, Italy, Latvia, and Slovenia as well. Nevertheless, after 180 days of vaccination, the US, Bulgaria, and Lithuania showed a slight increase in hospitalization rates.

### Vaccination vs. ICU

The analysis of the fully vaccinated populations vs. ICU patients per million is represented in Figure 4, where each bubble represents a country and the bubble size represents the population. The bubbles are color-coded by country. Prior to vaccination, the ICU patients per million were higher than 80 for Czechia, Slovenia, and the US. Initially, the vaccination rate was low for Czechia. After 60 days of vaccination, below 4% of the population was fully vaccinated, resulting in the ICU patient rate increasing drastically for Czechia; but after that, Czechia showed a drastic reduction in ICU patients (from 190 to 87.6) as the vaccination rate doubled. Immediately after the start of vaccination in Slovenia, ICU patients decreased, but after 60 days, the trend was impacted by the emergence of non-parental COVID-19 variants and ICU patients increased (Figure 4). Within a short span of 30 days, the increment in ICU patients came under control for Slovenia as the vaccination rate increased and ICU patients decreased simultaneously. For the first 60 days, after vaccination, the US showed a slight increment in the vaccination rate, and as expected the ICU patient rate declined accordingly. Thereafter, for the next 30 days, the ICU patient rate did not change much as the vaccination rate almost doubled (12.5–24.5) for the US. After 90 days of vaccination, except for Canada, as expected, the countries began moving to the left of the X-axis as the vaccination rates increased. Canada did not show a significant change in vaccination rate, and there was a slight increase in ICU patients for a short span of time, although later on, the vaccination rate increased, and the ICU admission of patients decreased. For Denmark, Finland, Ireland, Israel, Portugal, Spain, and the UK, the ICU patients per million were reduced to 20 (Figure 4). The reduction in ICU admission of patients continued and, after 120 days of vaccination, the ICU patients per million was reduced to 20 for Austria, Czechia, Estonia, Germany, Italy, Romania, Switzerland, and the US. After 150 days of vaccination, Bulgaria had the least vaccinated population but still the rate of ICU admissions was under 20. The rest of the countries had vaccination rates above 25 and ICU patient rates below 20. After 180 days of vaccination, ICU patient numbers showed an increment for the US even after over 40% of the population being fully vaccinated.

## Conclusion

COVID-19 outbreaks brought about significant global morbidity and mortality, as well as diminishing the economic and social well-being of individuals and communities. In spite of these devastating effects, the majority of the population remains susceptible to SARS-CoV-2 infection (34). Thus, vaccine development has been a high priority. The extent and speed of vaccine development efforts have been exceptional, and highly protective vaccines have been distributed all over the world. Our findings reveal that COVID-19 vaccines, even if they granted limited protection against infection, could essentially mitigate the future outbreaks, hospitalizations, and ICU admissions. However, it is necessary to further evaluate the success of vaccination programs over a long period of time to test their effectiveness in general. For our study, vaccination was only evaluated on the basis of confirmed cases. Even though the positive impact of vaccination in reducing in COVID-19 cases has been noted, exceptions were found for certain countries in certain time frames. In further studies, it is warranted to incorporate more variables, or for studies to be conducted with larger cohorts.

Given the limited population-level immunity to COVID-19 (35), vaccination plays a major role in providing preventive measures to reduce the disease burden and alleviate future attacks. Our study indicates that vaccination has a substantial impact on reducing the incidence, hospitalizations, and ICU admissions related to COVID-19. Our findings support the WHO recommendations (36), highlighting that a targeted vaccination strategy can efficiently mitigate disease burden and the economic impact of COVID-19. However, this impact is attained in the context of continued public health efforts and is not possible without a keen focus on the other aspects of infection prevention and control measures such as the use of masks, hand hygiene, testing, contact-tracing, and the isolation of infected cases. If current vaccination programs are accompanied by relaxation of other measures, much higher vaccine coverage will be necessary with a substantially higher distribution capability. Nonetheless, our findings are an encouraging signal of the potential benefits of vaccination against COVID-19.

## Data availability statement

Publicly available datasets were analyzed in this study. This data can be found at: https://ourworldindata.org/coronavirus.

## Author contributions

PS: Conceptualization, Data curation, Formal analysis, Investigation, Methodology, Software, Visualization, Writing – original draft. AA: Software, Writing – original draft, Validation. SR: Validation, Visualization, Writing – review & editing. AK: Validation, Visualization, Writing – review & editing. PG: Writing – review & editing. SK: Writing – review & editing. KG: Writing – review & editing, Supervision, Conceptualization, Formal analysis. HS: Supervision, Writing – review & editing, Funding acquisition, Project administration, Resources.

## Footnotes

^1^https://ccc.icmr.org.in/delta/positivity/

^2^https://ccc.icmr.org.in/delta/hospitalization/

^3^https://ccc.icmr.org.in/delta/icu/

^4^https://ccc.icmr.org.in/delta/delta/
